# Feasibility of a serious game system including a tangible object for post stroke upper limb rehabilitation: a pilot randomized clinical study

**DOI:** 10.3389/fneur.2023.1176071

**Published:** 2023-06-09

**Authors:** Samuel Pouplin, Céline Bonnyaud, Sylvain Bouchigny, Christine Mégard, Lucie Bertholier, Rafik Goulamhoussen, Pierre Foulon, Djamel Bensmail, Frédéric Barbot, Nicolas Roche

**Affiliations:** ^1^New Technologies Platform, Raymond-Poincaré Hospital, AP–HP, Université Paris Saclay, Garches, France; ^2^Paris-Saclay University, UVSQ, Research Unit ERPHAN, Versailles, France; ^3^Physiology and Functional Exploration Department, Raymond-Poincaré Hospital, AP–HP, Université Paris Saclay, Garches, France; ^4^CEA, LIST, Gif sur Yvette, France; ^5^Genious Healthcare, Groupe MindMaze, Montpellier, France; ^6^Physical and Rehabilitation Medicine Department, Raymond-Poincaré Hospital, AP–HP, Université Paris Saclay, Garches, France; ^7^End: icap Laboratory, Inserm Unit 1179, UVSQ, Montigny-le-Bretonneux, France; ^8^CIC 1429 INSERM, Raymond-Poincaré Hospital, AP–HP, Université Paris Saclay, Garches, France

**Keywords:** stroke, upper limb, rehabilitation, serious game, tangible object

## Abstract

**Introduction:**

Serious games can be used to provide intensive rehabilitation through attractive exercises as part of post-stroke rehabilitation. However, currently available commercial and serious games systems primarily train shoulder and elbow movements. These games lack the grasping and displacement components that are essential to improve upper limb function. For this reason, we developed a tabletop device that encompassed a serious game with a tangible object to rehabilitate combined reaching and displacement movements: the Ergotact system.

**Objectives:**

The aim of this pilot study was to assess the feasibility and the short-term effects of a training program using the Ergotact prototype in individuals with chronic stroke.

**Methods:**

Participants were assigned to one of two groups: a serious game training group (Ergotact) or a control training group (Self).

**Results:**

Twenty-eight individuals were included. Upper limb function increased after the Ergotact training program, although not statistically significantly, and the program did not induce pain or fatigue, demonstrating its safety.

**Conclusion:**

The Ergotact system for upper limb rehabilitation was well accepted and induced participant satisfaction. It complies with current recommendations for people with stroke to autonomously perform intensive active exercises in a fun context, in addition to conventional rehabilitation sessions with therapists.

**Clinical trial registration:**

https://clinicaltrials.gov/ct2/show/NCT03166020?term=NCT03166020&draw=2&rank=1, identifier NCT03166020.

## Introduction

Upper limb motor impairments affect 70% of people after stroke, reducing their participation in activities of daily living (1, 2). Improving upper limb function is therefore an essential goal of post-stroke rehabilitation (3). Current recommendations for rehabilitation include task-oriented training. For the upper limb, this involves training combined reaching, grasping, displacement and release actions (4). Training should be intense and include large numbers of repeated movements (5). Regular training should also be provided in the chronic phase of stroke to avoid degradation of motor capacities and functional abilities (6). However, a major issue over the long-term is maintaining patient motivation (7). The use of gaming technology has become popular as a complement to traditional rehabilitation approaches because of the fun, motivating and engaging nature of the games (8). Many studies have evaluated the use of commercially available systems designed for the general public, such as the Nintendo Wii, Microsoft Xbox Kinect or Sony PlayStation, for the rehabilitation of upper limb movements (9, 10). A systematic review of 19 studies that included a total of 215 individuals in the chronic phase of stroke showed that, despite the fact commercial games can be used to increase the intensity of upper limb practice, they have little impact on paretic upper limb function (11). According to a survey of 112 therapists, this is because of the lack of specificity of these games (12).

Serious games, designed specifically for rehabilitation, have been increasingly developed in recent years. These games can be used to provide intensive rehabilitation through attractive and fun exercises (11). Systematic reviews and meta-analyses have shown that, in contrast to commercial systems, serious games can improve the motor ability of the paretic upper limb after stroke (13). Furthermore, they may be more effective than conventional therapy, with a moderate pooled effect size for the same training duration (14). It is postulated that this greater efficacy is due to the motivational aspects of gaming, which stimulate patients to perform a greater number of repetitions (14). However, the currently available commercial and serious games systems primarily train shoulder and elbow movements and lack the grasping and displacement components that are essential to improve upper limb function (10, 15–17). Most studies have focused either on reaching or grasping, despite the fact these actions occur together during activities of daily living. In our opinion, it is essential to evaluate rehabilitation exercises that combine reaching and grasping movements, for example, during grasping and moving an object (18).

For this reason, we established a collaboration between engineers, developers and clinicians for the development of a tabletop device that encompassed a serious game with a connected, tangible object to rehabilitate combined reaching and displacement movements: the Ergotact system (19). Electronic tabletop games have a strong potential to facilitate the practice of specific and repeated movements, particularly if they involve the manipulation of tangible objects (20). For Ergotact, we chose a fantasy universe within the world of Kung Fu based on the results of a meta-analysis that showed that fantasy scenarios had the largest effect size compared to realistic, abstract and mixed scenarios: it is believed that fantasy characters and scenarios foster engagement (14). In the first phase of this project, the team of clinicians and engineers developed the touch table and tangible object as well as algorithmic rules for progression within the game.

Here we present the second phase of this project: a pilot study that aimed to assess the short-term effects of a training program using the Ergotact prototype on paretic upper limb impairment, function, pain, fatigue and quality of life in people with chronic stroke. Other data has been collected with the Ergotact prototype and a motion capture system (speed of the gesture to reach the target, kinematic, grip strength). We chose to focus here on the clinical approach and a first presentation of the device. We hypothesized that paretic upper limb function would increase and that the training program would not induce pain or fatigue.

## Methods

### Participants

Outpatients with chronic stroke followed in our university hospital were invited to participate in this study if they met the following inclusion criteria: (i) aged over 18 years; (ii) hemiparesis caused by a single hemispheric stroke more than 6 months previously; (iii) able to lift an object placed in the paretic hand from the table; (iv) able to understand instructions and the Ergotact program (aphasia severity score ≥ 3 points on the Boston Diagnostic Aphasia Examination); (v) having given written consent for participation in the study. People were not included if they had a Montreal Cognitive Assessment (MoCa) evaluation score < 17 (the score was checked against the patient’s medical record), points had undergone musculoskeletal surgery to the upper limb in the last 6 months, or had complex regional pain syndrome in the upper limb. The study was approved by an ethics committee and all participants provided written informed consent before participation. The study was registered prospectively on ClinicalTrials.gov (NCT03166020) and was supported by a French ANR grant (ANR-14-CE17-0019).

### Materials

**Figure 1:**
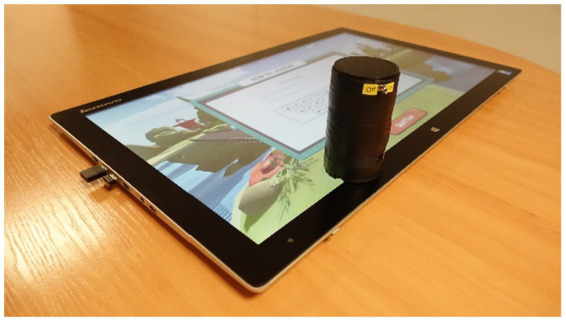
Ergotact prototype system.

The Ergotact prototype is shown in Figure 1. The tangible object was cylindrical in shape and was made using a custom-made 3D printing tool developed specifically for this project. The diameter of the cylinder (5 cm) was chosen to conform with the typical grasping capacity of people with chronic stroke (21). The shape of the tangible object also matched the shape of everyday life objects such as a glass or a cup. The serious game was displayed on a touch table that could support the movement of the tangible object.

The Ergotact game is based on a fantastic universe in which the player embodies a character training in kung fu, guided by his spiritual master. The character is moved by sliding the tangible object on the touch table, jumps by lifting the object, hits by squeezing the object, and hits using a stick by rotating the object (using wrist flexion or extension). At the beginning, the difficulty of the game was adjusted for each participant according to the results of the initial assessment on the touch table. During the game, a self-adaptive algorithm ensured that the participant always worked at the appropriate level. To progress to the next level of the game, the participant had to win a virtual battle otherwise they continued at the same level. If the participant could not progress to the next level, the game automatically adjusted the level of difficulty to match with the participant’s capacities.

A demonstration of the game is available here.^1^

### Experimental procedure

The short-term effects of training with Ergotact were compared with an upper-limb, home-based, self-rehabilitation program (22) which is currently proposed in our center to out-patients with chronic stroke.

Participants were assigned to one of the two groups: the serious game training group (Ergotact) or the control training group (Self). The randomization procedure was performed using computer-generated block randomization. Participants were randomly assigned to the study groups in a 1:1 ratio. Allocation was performed using sequentially numbered sealed opaque envelopes.

All participants continued their usual rehabilitation in non-specialized outpatient clinics.

The participants in the Ergotact group performed the 30-min training program in the center, supervised by a therapist. The Ergotact device was placed on the edge of a height adjustable table, centered on the participant’s sternum. The participant was seated in a chair without armrests, in front of the table, with their abdomen 5 cm from the edge. The trunk was constrained by a harness to promote paretic upper-limb movement rather than trunk compensations (23). The height of the table was adjusted so that the participant’s shoulder was flexed to 45° when the elbow was on the table.

Each training program was preceded by an assessment phase to adjust the level of the training to the participant’s capacities to avoid bias from heterogeneity of participant responses to the training. The training level was then set so that training objectives were 10% further than the movements performed during the assessment.

**Figure 2:**
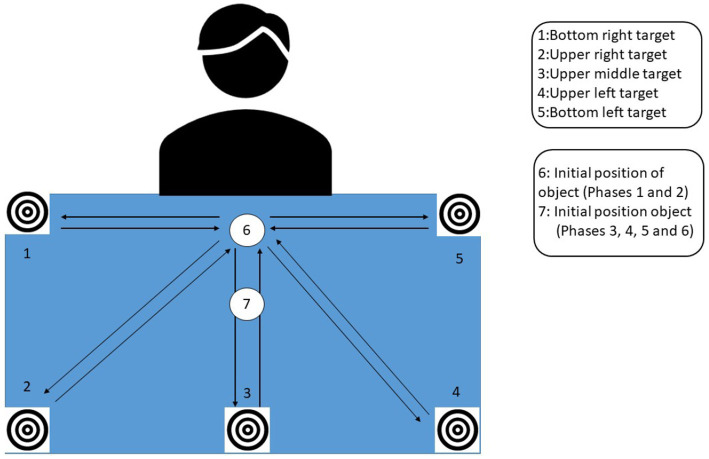
Touch table with the initial position of the tangible object and targets for the assessment.

Figure 2 presents the spatial configuration of the targets on the touch table for the assessment. The arrows correspond to the movements made by the participant.

The assessment lasted between 5 and 15 min depending on participant’s abilities. For phases 1 and 2, the participant grasped the object at position 6, which was the starting position for all the movements.

Phase 1: sliding the object from the bottom center of the table (position 6, Figure 2) to 5 targets that appeared at the different extremities of the tabletop: bottom right (position 5), upper right (position 4), upper middle (position 3), upper left (position 2), bottom left (position 1).

Phase 2: lifting the object from the bottom center of the table (position 6, Figure 2) to 5 targets that appeared at the different extremities of the tabletop (bottom left, upper left, upper middle, upper right, bottom right).

Phase 3: rotating the object to the right by performing wrist extension (for right-side hemiparesis) and vice versa for a left-side hemiparesis, at position 7 (Figure 2).

Phase 4: rotating the object to the left by performing wrist flexion (for right-side hemiparesis) and vice versa for a left-side hemiparesis, at position 7 (Figure 2).

Phase 5: squeezing the object as hard as possible and releasing, at position 7 (Figure 2).

Phase 6: lifting the object as high as possible, from position 7 (Figure 2).

The position of the targets induced the movement direction; targets were positioned both on the paretic side (to stimulate shoulder abduction) and on the non-paretic side, and in the front of the workspace (to stimulate elbow extension) and the back of the workspace. The results of the assessments defined the participant’s workspace and the dimensions of the game display were adjusted to the participant’s motor capacity.

The target on the top of the touch table on the paretic side involved shoulder flexion/abduction and possibly external rotation and elbow extension. The target on the top of the touch table on the non-paretic side involved shoulder flexion/adduction and possibly internal rotation and elbow extension. The target on the bottom of the touch table on the paretic side involved shoulder abduction and possibly external rotation, and elbow flexion. The target on the bottom of the touch table on the non-paretic side involved shoulder adduction and possibly internal rotation and elbow extension. Some tasks required wrist flexion/extension and others required grasping / releasing of the object.

The participants in the self-training group (SELF) performed self-rehabilitation at home. During the inclusion visit in the center, they participated in a 30-min session with a therapist who taught them the self-rehabilitation program. The exercises focused on upper-limb impairments and functional limitations. They included 3 stretching exercises, 3 strengthening exercises and 3 specific task-oriented exercises. The stretching exercises targeted the shoulder adductors, elbow flexors and wrist flexors. The strengthening exercises involved lifting an object as high as possible, extending the elbow, and performing wrist extension. The task-oriented exercises involved grasping and moving a bottle, grasping and moving a glass, and holding a bottle of water with the paretic hand while opening it with the other hand. These exercises have been described previously (22). A booklet with photographs and detailed instructions of each exercise was provided to the participants.

The training duration was the same for both groups: 30 min per session, 5 days a week, for 2 weeks (10 sessions). This frequency and duration was chosen to ensure that the protocol was acceptable for participants and because we did not find evidence in the literature that the duration of the training would influence effectiveness (14). Moreover, in view of the fact very specific movements were repeatedly trained, in particular with the Ergotact, we expected short-term improvements to occur in paretic upper limb function. In addition, the duration was constrained by the fact we had only one prototype.

### Outcomes

Outcomes were evaluated in both groups at day 0 (D0: before the training) and at day 15 (D15: at the end of the training sessions). The occupational therapist who performed the measures was blinded to group assignment.

### Outcome measures

#### Assessment of impairment

- *Upper extremity motor impairments* were assessed using the Upper Limb Fugl Meyer scale, which is composed of 22 items and yields an overall score ranging from 0 (worst) to 66 (normal) (24).
- *Spasticity* of the elbow, wrist and finger flexors was evaluated using the Modified Ashworth Scale (MAS) (25).
- *Pain* during upper limb movement was evaluated on a ten-point visual analog scale (0: “no pain” to 10: “unbearable pain”).
- *Fatigue following the training sessions* was assessed with the Fatigue Severity Scale (26).

#### Assessment of function

- *Upper limb motor and functional capacity* was assessed using the Wolf Motor Function Test. The maximum time allowed per task is 120 s and performance is rated on a 6-point scale (of 0–5) where 0 = inability to perform the task and 5 = performance of the task is similar to a healthy subject (27). Change in proximal (first 6 items) and distal (last 9 items) performance time and score were also analyzed.
- *Gross manual dexterity* was evaluated using the Box and block test. It consists of grasping, moving and releasing as many 2.5-cm cubes as possible from one box to another in 1 min (28).
- *Complex activities in different categories of daily living* were assessed using the Frenchay activities Index. It includes 15 items, and the score is based on the frequency at which activities are performed in daily life ranging from 0 (inactive) to 45 (very active) (29).

#### Quality of life

- *Quality of life* was assessed with the Short Form Health Survey SF12 (30).

#### Satisfaction/motivation

- *Satisfaction* and *motivation* were each assessed on a ten-point visual analog scale (0: “worst” to 10: “best”). At the baseline assessment (D0), participants were asked to evaluate their satisfaction with their usual conventional rehabilitation in a non-specialized outpatient clinic and their level of motivation to continue. At D15, they were asked to evaluate their satisfaction with either the Ergotact or self-rehabilitation program and their level of motivation to continue.

### Data analysis

Descriptive statistics were calculated and data are presented as medians (interquartile ranges) for continuous variables and frequencies for categorical variables. Because of the explanatory design of this study and the small sample, non-parametric tests were used. Differences between the two groups in age and time since lesion were compared using the Mann Whitney U test and differences between sex, laterality, type of stroke and side of hemiparesis, were compared using the Fisher exact test. The Wilcoxon test was used to evaluate changes in outcomes between D0 and D15 for each group. Between group differences in change were evaluated using the Mann–Whitney U test. Intention-to-treat analysis was performed, and missing data were imputed with the Last Observation Carried Forward LOCF method. The level of significance was fixed at *p* < 0.05. Data were analyzed using R software version 3.6.1.

## Results

**Figure 3:**
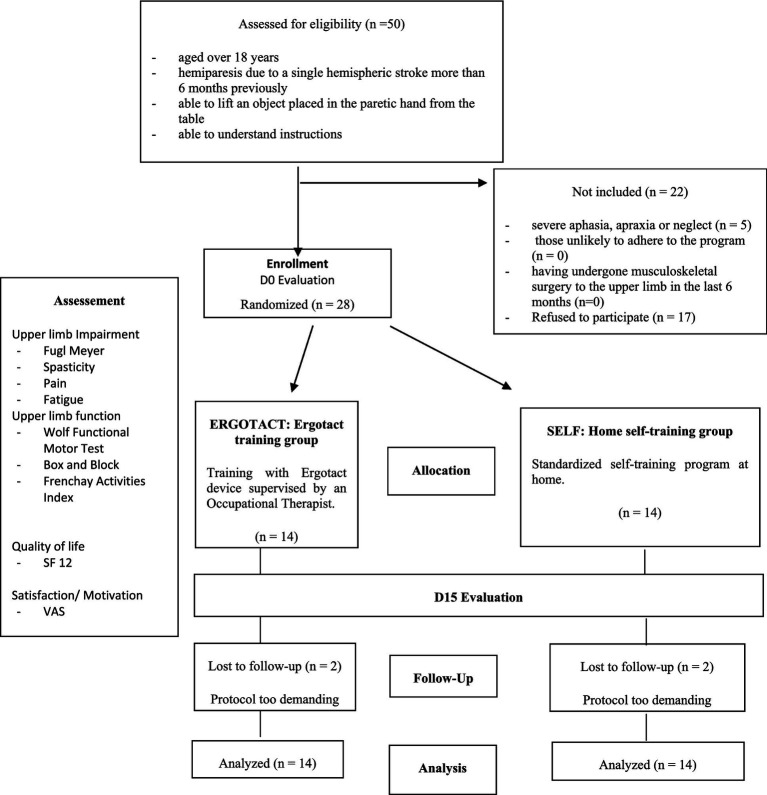
Study flow chart.

Of the 50 eligible participants invited to take part in the study, 17 could not participate because their professional activity or their schedule was not compatible with the protocol requirements (i.e., daily attendance at the center if allocated to the Ergotact group). Five met an exclusion criterion, thus 28 were included (13 females and 15 males, mean ± SD age 61.8 ± 13.5 years). Four dropped out during the protocol thus 24 participants completed the study. Figure 3 shows the trial flow chart.

Participant characteristics are shown in Table 1.

**Table 1:** Demographic characteristics of participants.

	Ergotact			Self			*p*
	*N*	Mean	SD	*N*	Mean	SD	
Sex							0.45
Male	6			9			
Female	8			5			
Age (years)		55.8	15.7		67.8	7.5	0.03
Time since lesion (years)		12.5	11.8		9.42	4.7	0.52
Laterality							1.00
Right-handed	12			12			
Left-handed	1			2			
Ambidextrous	1			0			
Type of stroke							0.67
Hemorrhagic	3			5			
Ischemic	11			9			
Side of hemiparesis							0.25
Right	8			4			
Left	6			10			
MOCA score		23.71	3.38		24.00	3.111	0.64

Age, time since lesion: Mann Whitney test. Sex, laterality, type of stroke and side of hemiplegia: Fisher exact test.

There were no differences between the Ergotact and Self groups for sex (*p* = 0.45), time since lesion (*p* = 0.52), laterality (*p* = 1), type of stroke (*p* = 0.67), side of hemiplegia (*p* = 0.25) or Moca Score (*p* = 0.64). Participants in the Ergotact group were younger than those in the Self group (*p* = 0.03).

### Impairment and function

Table 2 shows the results of the upper limb impairment and function tests.

**Table 2:** Results for the impairment and function tests (Fugl Meyer, upper limb pain, fatigue severity scale, WFMT, box and block, Frenchay activities index).

	Ergotact		Ergotact *p* (D15 vs D0)	Self		Self *p* (D15 vs D0)	*p* (Ergotact vs Self)
	Median D0 [IQR]	Median D15 [IQR]		Median D0 [IQR]	Median D15 [IQR]		
Impairment assessment							
Fugl-Meyer							
Fugl-Meyer	39.50 [22.00; 55.75]	40.00 [28.75; 54.25]	0.68	46.00 [35.00; 51.00]	46.50 [44.38; 53.12]	0.97	0.20
Modified Ashworth scale score for each muscle group							
Elbow F	1.50 [1.00; 1.88]	1.25 [1.00; 2.00]	0.68	1.00 [1.00; 1.00]	1.00 [1.00; 1.00]	0.68	1.00
Wrist F	1.00 [0.00; 1.50]	1.50 [1.38; 2.00]	0.05	1.00 [0.00; 1.00]	0.50 [0.00; 1.12]	0.34	0.20
MP F	1.00 [0.00; 1.50]	1.50 [0.00; 1.50]	0.35	0.50 [0.00; 1.50]	0.50 [0.00; 1.50]	0.37	0.54
FDS	0.00 [0.00; 0.00]	0.00 [0.00; 1.12]	0.09	0.00 [0.00; 1.00]	0.00 [0.00; 1.00]	1.00	0.07
FDP	0.00 [0.00; 0.00]	0.00 [0.00; 1.00]	0.17	0.00 [0.00; 0.00]	0.00 [0.00; 0.00]	1.00	0.09
Upper limb pain							
Pain during movement	1.50 [0.12; 5.38]	0.30 [0.00; 3.25]	0.34	1.00 [0.00; 3.50]	0.30 [0.00; 3.65]	0.93	0.35
Fatigue Severity Scale							
Fatigue Severity Scale	4.39 [2.69; 5.36]	4.00 [3.58; 5.83]	0.98	3.89 [2.56; 4.33]	3.67 [3.06; 4.25]	0.61	0.98
Motor function of the upper limb assessment							
WFMT/box and block/Frenchay activities index							
WFMT time	423.00 [211.00; 776.00]	465.00 [273.00; 683.00]	1.00	215.00 [88.00; 833.00]	327.00 [69.00; 569.00]	1.00	0.84
WFMT score	36.00 [20.22; 52.00]	37.00 [24.25; 49.50]	0.76	41.00 [31.00; 46.0]	42.19 [33.75; 46.50]	0.87	0.71
WFMTp time	14.98 [11.73; 25.41]	16.30 [9.98; 34.31]	0.13	13.25 [10.67; 22.27]	14.77 [8.38; 18.12]	0.85	0.24
WFMTp score	17.00 [12.00; 24.50]	19 [10.75; 23.25]	0.44	19.00 [16.00; 21.00]	18.50 [17.75; 21.25]	0.90	0.46
WFMTd time	386.00 [186.00; 696.00]	370.00 [260.00; 543.00]	1.00	199.00 [75.00; 567]	270.00 [56.00; 519.00]	0.85	0.84
WFMTd score	19.00 [9.00; 26.25]	19.00 [9.00; 26.25]	0.94	23.00 [17.00; 25.00]	22.19 [16.75; 25.81]	0.86	0.58
Box and Block	11.50 [0.25; 19.50]	8.00 [0.75; 17.25]	0.72	14.50 [3.00; 26.25]	10.50 [3.75; 24.25]	0.65	0.48
FAI Total	24.00 [21.25; 32.25]	26.00 [15.50; 31.25]	0.76	14.00 [8.00; 17.00]	18.50 [13.00; 27.00]	0.03*	0.23
FAI domestic chores	10.00 [5.25; 12.00]	9 [6.00; 14.00]	*p* = 0.67	4.00 [0.00; 6.00]	6.00 [0.75; 9.00]	0.06	0.61
FAI leisure/work	7.00 [4.00; 9.00]	5.50 [4.00; 7.50]	*p* = 0.83	3.00 [2.00; 5.00]	4.50 [2.00; 5.50]	0.10	0.37
FAI outdoor activities	10.50 [7.50; 11.75]	9.5 [7.5; 17.5]	*p* = 0.84	7.00 [5.00; 10.00]	11.00 [7.50; 12.25]	0.04*	0.13

Differences between D0 and D15 for each group: paired Wilcoxon test. Between, group differences in change: Mann–Whitney *U* test. MP, metacarpal phalangeal; F, flexors; FDS, flexor digitorum superficialis; FDP, flexor digitorum profundus; VAS, visual analog scale WMFTp, proximal section of WMFT items 1–6; WMFTd, distal section of WMFT items 8–13 and 15; FAI, Frenchay activities index; *, intragroup significant difference.

There were no within-group differences between the baseline and end of training outcomes in either group for the Fugl Meyer, the modified Ashworth scale, the Fatigue Severity Scale, the WFMT, the Box and Block test or pain during movement. The total Frenchay activities index score and the outdoor activities dimension score increased significantly only in the SELF group.

### Quality of life, satisfaction and motivation

Table 3 shows the results of the SF12 Physical health score, SF12 Mental Health score, and satisfaction and motivation ratings.

**Table 3:** Results of the SF-12, satisfaction and motivation questionnaires.

	Ergotact		Ergotact *p* (V2 vs V1)	Self		Self *p* (V2 vs V1)	*p* (Ergotact vs Self)
	Median D0 [IQR]	Median D15 [IQR]		Median D0 [IQR]	Median D15 [IQR]		
SF-12							
SF-12 (PCS score)	32.89 [30.97; 38.16]	33.54 [23.15; 37.53]	0.91	30.38 [2.56; 4.33]	32.49 [27.75; 35.97]	0.91	0.63
SF-12 (MCS score)	43.21 [39.03; 49.67]	45.85 [41.47; 51.29]	0.47	49.39 [36.80; 54.03]	48.71 [43.57; 58.99]	0.47	0.51
Satisfaction/motivation regarding intervention							
Satisfaction VAS	6.00 [3.88; 9.62]	8.00 [5.07; 8.62]	0.37	5.00 [3.10; 8.00]	7.10 [4.43; 8.27]	0.37	0.62
Motivation VAS	8.1 [7.08; 9.18]	8.25 [7.72; 9.85]	0.9	9.00 [5.75; 10.00]	8.25 [7.05; 10.00]	0.83	0.97

Differences between D0 and D15 for both groups: paired Wilcoxon test. Between group difference: Mann–Whitney *U* test. PCS, physical health score; MCS, mental health score; VAS, visual analogic scale.

There were no significant within-group differences between the baseline and end of training measures in either group for quality of life (SF12 Physical health score, SF12 Mental Health score), satisfaction or motivation.

## Discussion

This pilot study assessed the short-term effects of training sessions using the Ergotact system, a prototype serious gaming system developed to rehabilitate object displacement in individuals with chronic stroke. As hypothesized, upper limb function increased following the Ergotact training program, although not statistically significantly, and the program did not induce pain or fatigue, demonstrating its safety.

The improvements in certain outcomes are interesting and encouraging. We analyzed the proximal and distal WMFT scores and time separately since stroke often causes differences in motor capacity between these limb segments. Although the between-group differences were not statistically significant, more improvements occurred in the Ergotact group: the proximal score and performance time increased, the distal score did not change and, the distal time decreased; in contrast, in the self-group, all the scores decreased, and all the performance times increased. The fact that in the Ergotact group the improvement in some scores was associated with a concomitant increase in performance times suggests an interaction between speed and ability for the performance of functional tasks: participants may have performed movements more slowly in order to generate better movements.

An important result of this study is that it highlighted the difficulty improving upper limb function in the chronic phase of stroke. The mean time since stroke onset in the whole sample was 10.97 (8.99) years. By this time, it is likely that substantial physiological and anatomical changes will have occurred within the muscles and joints. Furthermore, individuals will likely have accommodated to their disability by developing habits of function that are difficult to alter, compared with those in the first or second year of their stroke.

Two weeks seems a too short period for the rehabilitation of the paretic upper-limb of people with stroke, and may be the reason for the non-significant improvement in both groups. Although the meta-analysis by Tǎut et al. (14) did not find any effect of the duration of serious game training on the results, consensus within the scientific community advocates for longer durations of rehabilitation (14). A study by our group on the effects of the same upper limb self-rehabilitation program performed in addition to botulinum toxin injections (30 min daily for 4 weeks) also found only weak effects of self-rehabilitation (30). However, in that study, participants performed more self-rehabilitation than prescribed, indicating that they felt it was worth it. A recent large-scale study showed that significant changes occurred after a 90-h program (31). Therefore, we suggest that future studies focus on upper limb rehabilitation programs of longer duration and/or intensity for chronic stroke.

Despite the randomized allocation of participants to the two groups, at baseline the Fugl-Meyer score was much lower in the Ergotact group than in the Self group, although the difference was not significant. Some participants in the Ergotact group had severe impairment that may have reduced their chances of improving in the short study timeframe. Likewise, the participants in the Self group were significantly older than those in the Ergotact group, which may have affected the results of the study. It could be hypothesized that older participants would adhere less to the gaming nature of the Ergotact system, however another study found no effect of age on adhesion (31).

Other factors intrinsic to the Ergotact games could have affected the results. Determining the appropriate level of compromise between the difficulty of the game (target size and sensitivity of the target validation, for example) to ensure the participant is challenged, while allowing them to achieve the goals to maintain their motivation (8) is highly complex. This aspect may require further exploration of the self-adaptive algorithm in order to optimize the Ergotact games.

Importantly, the Ergotact training program did not induce fatigue, upper limb pain or increase spasticity, demonstrating the safety of the exercises. Furthermore, the level of satisfaction and motivation to pursue this type of training were high. The level of motivation relating to Ergotact training was slightly higher than for usual, non-specialized out-patient rehabilitation, while for the self-training, it was slightly lower. This is consistent with data in the literature that indicate that video gaming based on fantastic scenarios is motivating (11, 14). The higher level of participant satisfaction with Ergotact compared with usual, non-specialized out-patient rehabilitation is in line with the opinions of therapists collected in a previous, ergonomic study of Ergotact: therapists reported that the Ergotact system fulfilled an unmet need for chronic upper limb stroke rehabilitation (19). Nevertheless, the 2 weeks of rehabilitation were too short to highlight a significant difference between the motivation and satisfaction of the two groups. A longer-term study is required to determine if the high motivation and satisfaction scores persist over time.

The Ergotact system as a rehabilitation tool meets current recommendations regarding the provision of intensive exercises for people with stroke. Because of the exploratory nature of this study, the Ergotact training was performed under therapist supervision, however, the system could be used for self-rehabilitation in addition to supervised rehabilitation sessions, thus increasing rehabilitation intensity (11). Despite the fact the cost is somewhat higher than a simple exercise-based self-rehabilitation program, the Ergotact system opens more prospects for home rehabilitation, with a self-adaptive algorithm to ensure that the individual always works at the appropriate level. Furthermore, training with digital technology allows the possibility to transfer data to the rehabilitation center for follow-up and support by a therapist, which is recommended in the chronic phase of stroke (32, 33). Providing continued support to exercise is important to prevent functional deterioration (6). The Ergotact system was developed to be relatively low-cost and based on a tablet-like device which is familiar to the general public, and thus easy to use in the person’s home. Furthermore, play promotes motivation and involvement (14). The Ergotact system also offers many long-term perspectives, such as playing within a network of other individuals with stroke, or a multiplayer mode in which the family could play together. Such functions could help to maintain long-term motivation and increase home rehabilitation time.

The Ergotact prototype provides a new, objective assessment of reaching and grasping capacity after stroke and an innovative serious game that meets the recommendations for intensive motor training after stroke (in a context of limited therapist time). To our knowledge, there is currently no rehabilitation device that simultaneously trains reaching and grasping. In addition, the Ergotact prototype allows the individual to training autonomously outside of conventional rehabilitation sessions.

### Limitations

The main limitation of this study was that the duration of training chosen was too short. However, this study was only the second step in the development and evaluation process of the Ergotact system after the ergonomic study by Mégard et al. (19). The study generated preliminary data that are required prior to performing a large randomized, controlled trial, for example to calculate the sample size. We performed a sample size calculation using our results, but it showed that more than one thousand participants would be necessary. Therefore, we suggest that future studies should evaluate rehabilitation programs of at least 1 month in duration (34, 35) with more intense rehabilitation (at least 3 h per day) (36); this would be consistent with the objective of the Ergotact prototype.

## Conclusion

The prototype Ergotact system developed through a collaboration between engineers, developers and clinicians trains object displacement concomitantly with upper limb movements in a large workspace. The high participant ratings of motivation and satisfaction demonstrate the pertinence of its use in addition to conventional rehabilitation sessions. Furthermore, the training had no adverse effects. The prototype Ergotact provides new technological possibilities for upper limb rehabilitation. It complies with current recommendations for the autonomous performance of intensive active exercises in a playful context, in addition to conventional rehabilitation sessions with therapists. These preliminary data justify the performance of a large, randomized, controlled trial involving daily, unsupervised home-based training sessions over a very long period of time to determine the effectiveness of the Ergotact system in improving upper limb function, and to evaluate long-term adherence to training using such a system.

## Data availability statement

The raw data supporting the conclusions of this article will be made available by the authors, without undue reservation.

## Ethics statement

The studies involving human participants were reviewed and approved by the N°IDRCB: 2016-A01903-48 ANSM Agence Nationale Sécurité Médicament et Produits de Santé. The patients/participants provided their written informed consent to participate in this study.

## Author contributions

SP, CB, SB, CM, LB, RG, PF, DB, FB, and NR substantial contributed to the conception and design of the manuscript. SP, CB, and NR contributed to acquisition, analysis, and interpretation of the data. SP revised it critically. All authors participated to drafting the manuscript, contributed equally to the manuscript, and read and approved the final version of the manuscript.

## Funding

This study was supported by a grant from ANR (agreement number: ANR-14-CE17-0019).

## Conflict of interest

The authors declare that the research was conducted in the absence of any commercial or financial relationships that could be construed as a potential conflict of interest.

## Publisher’s note

All claims expressed in this article are solely those of the authors and do not necessarily represent those of their affiliated organizations, or those of the publisher, the editors and the reviewers. Any product that may be evaluated in this article, or claim that may be made by its manufacturer, is not guaranteed or endorsed by the publisher.

## Footnotes

^1^https://www.youtube.com/watch?v=uA9ttrZfg_Q&ab_channel=GENIOUSHealthcare

## References

[1] Kwakkel G Kollen BJ van der Grond J Prevo AJH. Probability of regaining dexterity in the flaccid upper limb: impact of severity of paresis and time since onset in acute stroke. Stroke. (2003) 34:2181–6. doi: 10.1161/01.STR.0000087172.16305.CD, PMID: 12907818

[2] Raghavan P. Upper limb motor impairment after stroke. Phys Med Rehabil Clin N Am. (2015) 26:599–610. doi: 10.1016/j.pmr.2015.06.008, PMID: 26522900 PMC4844548

[3] Carlsson H Gard G Brogårdh C. Upper-limb sensory impairments after stroke: self-reported experiences of daily life and rehabilitation. J Rehabil Med. (2018) 50:45–51. doi: 10.2340/16501977-2282 29068038

[4] Rensink M Schuurmans M Lindeman E Hafsteinsdóttir T. Task-oriented training in rehabilitation after stroke: systematic review. J Adv Nurs. (2009) 65:737–54. doi: 10.1111/j.1365-2648.2008.04925.x, PMID: 19228241

[5] Oujamaa L Relave I Froger J Mottet D Pelissier JY. Rehabilitation of arm function after stroke. Literature review. Ann Phys Rehabil Med. (2009) 52:269–93. doi: 10.1016/j.rehab.2008.10.003 19398398

[6] Dhamoon MS Longstreth WT Bartz TM Kaplan RC Elkind MSV. Disability trajectories before and after stroke and myocardial infarction the cardiovascular health study. JAMA Neurol. (2017) 74:1439–45. doi: 10.1001/jamaneurol.2017.2802, PMID: 29059266 PMC5772778

[7] Rijken PM Dekker J. Clinical experience of rehabilitation therapists with chronic diseases: a quantitative approach. Clin Rehabil. (1998) 12:143–50. doi: 10.1191/026921598669374346, PMID: 9619656

[8] Delbressine F Timmermans A Beursgens L De Jong M Van Dam A Verweij D. Motivating arm-hand use for stroke patients by serious games. Proc. Conf IEEE Eng Med Biol Soc. (2012) 2012:3564–7. doi: 10.1109/EMBC.2012.6346736, PMID: 23366697

[9] Aramaki AL Sampaio RF Reis ACS Cavalcanti A Dutra FCMSE. Virtual reality in the rehabilitation of patients with stroke: an integrative review. Arq Neuropsiquiatr. (2019) 77:268–78. doi: 10.1590/0004-282x20190025, PMID: 31090808

[10] Ain QU Khan S Ilyas S Yaseen A Tariq I Liu T. Additional effects of Xbox Kinect training on upper limb function in chronic stroke patients: a randomized control trial. Healthcare. (2021) 9:242. doi: 10.3390/healthcare9030242 33668355 PMC7996301

[11] Thomson K Pollock A Bugge C Brady M. Commercial gaming devices for stroke upper limb rehabilitation: a systematic review. Int J Stroke Juin. (2014) 9:479–88. doi: 10.1111/ijs.12263 24661797

[12] Thomson K Pollock A Bugge C Brady MC. Commercial gaming devices for stroke upper limb rehabilitation: a survey of current practice. Disabil Rehabil Assist Technol. (2015) 30:1–8. doi: 10.3109/17483107.2015.1005031 25634339

[13] Proença JP Quaresma C Vieira P. Serious games for upper limb rehabilitation: a systematic review. Disabil Rehabil Assist Technol. (2018) 13:95–100. doi: 10.1080/17483107.2017.1290702 28359181

[14] Di T Pintea S Roovers JPWR Mañanas MA Bǎban A. Play seriously: effectiveness of serious games and their features in motor rehabilitation. A meta-analysis. NeuroRehabilitation. (2017) 41:105–18. doi: 10.3233/NRE-171462 28527226

[15] Kong KH Loh YJ Thia E Chai A Ng CY Soh YM. Efficacy of a virtual reality commercial gaming device in upper limb recovery after stroke: a randomized, controlled study. Top Stroke Rehabil. (2016) 23:333–40. doi: 10.1080/10749357.2016.1139796 27098818

[16] Lee G. Effects of training using video games on the muscle strength, muscle tone, and activities of daily living of chronic stroke patients. J Phys Ther Sci. (2013) 25:595–7. doi: 10.1589/jpts.25.595, PMID: 24259810 PMC3804992

[17] Choi JH Han EY Kim BR Kim SM Im SH Lee SY. Effectiveness of commercial gaming-based virtual reality movement therapy on functional recovery of upper extremity in subacute stroke patients. Ann Rehabil Med. (2014) 38:485–93. doi: 10.5535/arm.2014.38.4.485, PMID: 25229027 PMC4163588

[18] Woldag H Stupka K Hummelsheim H. Repetitive training of complex hand and arm movements with shaping is beneficial for motor improvement in patients after stroke. J Rehabil Med. (2010) 42:582–7. doi: 10.2340/16501977-0558, PMID: 20549164

[19] Megard C Bouchigny S Martin A Goulamhoussen R Bertholier L Foulon P. Ergotact: including force-based activities into post-stroke rehabilitation In: Extended abstracts of the 2019 CHI conference on human factors in computing systems. Glasgow, Scotland, UK: ACM (2019). 1–6.

[20] Li Y Fontijn W Markopoulos P. A tangible tabletop game supporting therapy of children with cerebral palsy. In: Markopoulos P Ruyter B De Ijsselsteijn W Rowland D. Fun and games. Berlin, Heidelberg: Springer; (2008). p. 182–193. (Lecture Notes in Computer Science; vol. 5294).

[21] García Álvarez A Roby-Brami A Robertson J Roche N. Functional classification of grasp strategies used by hemiplegic patients. PLoS One. (2017) 12:e0187608. doi: 10.1371/journal.pone.0187608 29125855 PMC5695285

[22] Bonnyaud C Gallien P Decavel P Marque P Aymard C Pellas F. Effects of a 6-month self-rehabilitation programme in addition to botulinum toxin injections and conventional physiotherapy on limitations of patients with spastic hemiparesis following stroke (ADJU-TOX): protocol study for a randomised controlled, investigator blinded study. BMJ Open. (2018) 8:e020915. doi: 10.1136/bmjopen-2017-020915, PMID: 30166290 PMC6119443

[23] Michaelsen SM Dannenbaum R Levin MF. Task-specific training with trunk restraint on arm recovery in stroke: randomized control trial. Stroke. (2006) 37:186–92. doi: 10.1161/01.STR.0000196940.20446.c9, PMID: 16339469

[24] Gladstone DJ Danells CJ Black SE. The fugl-meyer assessment of motor recovery after stroke: a critical review of its measurement properties. Neurorehabil Neural Repair. (2002) 16:232–40. doi: 10.1177/154596802401105171, PMID: 12234086

[25] Gregson J. Reliability of measurement of muscle tone and muscle power in stroke patients. Age Ageing. (2000) 29:223–8. doi: 10.1093/ageing/29.3.223, PMID: 10855904

[26] Cumming TB Packer M Kramer SF English C. The prevalence of fatigue after stroke: a systematic review and meta-analysis. Int J Stroke. (2016) 11:968–77. doi: 10.1177/1747493016669861 27703065

[27] Nijland R Van Wegen E Verbunt J Van Wijk R Van Kordelaar J Kwakkel G. A comparison of two validated tests for upper limb function after stroke: the wolf motor function test and the action research arm test. J Rehabil Med. (2010) 42:694–6. doi: 10.2340/16501977-0560, PMID: 20603702

[28] Chen HM Chen CC Hsueh IP Huang SL Hsieh CL. Test-retest reproducibility and smallest real difference of 5 hand function tests in patients with stroke. Neurorehabil Neural Repair. (2009) 23:435–40. doi: 10.1177/1545968308331146, PMID: 19261767

[29] Monteiro M Maso I Sasaki AC Barreto Neto N Oliveira Filho J Pinto EB. Validation of the Frenchay activity index on stroke victims. Arq Neuropsiquiatr. (2017) 75:167–71. doi: 10.1590/0004-282x20170014 28355324

[30] Bohannon RW Maljanian R Lee N Ahlquist M. Measurement properties of the short form (SF)-12 applied to patients with stroke. Int J Rehabil Res. (2004) 27:151–4. doi: 10.1097/01.mrr.0000127349.25287.de 15167114

[31] Kwakkel G Wagenaar RC Kollen BJ Lankhorst GJ. Predicting disability in stroke--a critical review of the literature. Age Ageing. (1996) 25:479–89. doi: 10.1093/ageing/25.6.479, PMID: 9003886

[32] Laver KE Lange B George S Deutsch JE Saposnik G Crotty M. Virtual reality for stroke rehabilitation. Cochrane Database Syst Rev. (2017) 11:CD008349. doi: 10.1002/14651858.CD008349.pub4, PMID: 29156493 PMC6485957

[33] Laffont I Froger J Jourdan C Bakhti K van Dokkum LEH Gouaich A. Rehabilitation of the upper arm early after stroke: video games versus conventional rehabilitation. A randomized controlled trial. Ann Phys Rehabil Med. (2020) 63:173–80. doi: 10.1016/j.rehab.2019.10.009, PMID: 31830535

[34] Maulet T Pouplin S Bensmail D Zory R Roche N Bonnyaud C. Self-rehabilitation combined with botulinum toxin to improve arm function in people with chronic stroke. A randomized controlled trial. Ann Phys Rehabil Med. (2021) 64:101450. doi: 10.1016/j.rehab.2020.10.004 33152520

[35] Harris JE Eng JJ Miller WC Dawson AS. A self-administered graded repetitive arm supplementary program (GRASP) improves arm function during inpatient stroke rehabilitation: a multi-site randomized controlled trial. Stroke. (2009) 40:2123–8. doi: 10.1161/STROKEAHA.108.544585 19359633

[36] Niama Natta DD Alagnide E Kpadonou GT Stoquart GG Detrembleur C Lejeune TM. Feasibility of a self-rehabilitation program for the upper limb for stroke patients in Benin. Ann Phys Rehabil Med. (2015) 58:322–5. doi: 10.1016/j.rehab.2015.08.003, PMID: 26419296

